# Food-provisioning negatively affects calf survival and female reproductive success in bottlenose dolphins

**DOI:** 10.1038/s41598-019-45395-6

**Published:** 2019-06-20

**Authors:** V. Senigaglia, F. Christiansen, K. R. Sprogis, J. Symons, L. Bejder

**Affiliations:** 1 Aquatic Megafauna Research Unit, Centre for Sustainable Aquatic Ecosystems, Harry Butler Institute, Murdoch University, Murdoch, 6150 Australia; 2 Environmental and Conservation Sciences, Murdoch University, Murdoch, 6150 Australia; 3 Aarhus Institute of Advanced Studies, Høegh-Guldbergs Gade 6B, Aarhus, 8000 Denmark; 4 Zoophysiology, Department of Bioscience, Aarhus University, Aarhus, 8000 Denmark; 5 Hawaii Institute of Marine Biology, University of Hawaii at Manoa, Kaneohe, HI 96744 USA

**Keywords:** Conservation biology, Behavioural ecology

## Abstract

Food-provisioning of wildlife can facilitate reliable up-close encounters desirable by tourists and, consequently, tour operators. Food-provisioning can alter the natural behavior of an animal, encouraging adverse behavior (e.g. begging for food handouts), and affect the reproductive success and the viability of a population. Studies linking food-provisioning to reproductive success are limited due to the lack of long-term datasets available, especially for long-lived species such as marine mammals. In Bunbury, Western Australia, a state-licensed food-provisioning program offers fish handouts to a limited number of free-ranging bottlenose dolphins (*Tursiops aduncus*). Coupled with long-term historical data, this small (<200 individuals), resident dolphin population has been extensively studied for over ten years, offering an opportunity to examine the effect of food-provisioning on the reproductive success of females (*n*_total_ = 63; *n*_provisioned females_ = 8). Female reproductive success was estimated as the number of weaned calves produced per reproductive years and calf survival at year one and three years old was investigated. The mean reproductive success of provisioned and non-provisioned females was compared using Bayes factor. We also used generalized linear models (GLMs) to examine female reproductive success in relation to the occurrence of food-provisioning, begging behavior and location (within the study area). Furthermore, we examined the influence of these variables and birth order and climatic fluctuations (e.g. El Niño Southern Oscillation) on calf survival. Bayes factor analyses (Bayes factor = 6.12) and results from the best fitting GLMs showed that female reproductive success and calf survival were negatively influenced by food-provisioning. The negative effects of food-provisioning, although only affecting a small proportion of the adult females’ population (13.2%), are of concern, especially given previous work showing that this population is declining.

## Introduction

With an increase in the human population that reside in cities, nature-based-tourism, particularly involving charismatic megafauna, is becoming a widespread and popular phenomenon to reconnect with nature^1^. Short-term benefits for people interacting with wildlife have been well-documented^2,3^ and encompass physiological, psychological and social gains such as relief from stress, enjoyment and a sense of accomplishment^4^. Close-up wildlife encounters can stimulate strong emotional responses in humans which, in turn, can inspire conservation action and provoke long-term behavioral changes^5,6^. Moreover, nature-based tourism also brings great financial rewards. For example, in 2009, whale and dolphin watching was a $2 billion industry employing 13,000 people and engaging >13 million tourists^7,8^.

Food rewards can facilitate and promote close-up encounters between people and wildlife^9^ and the tourism industry capitalizes on this practice to promote reliable access to animals at wildlife-watching locations^10^. However, intentional feeding of free-ranging animals is a contentious issue^10^ and the potential impacts on targeted populations must be carefully considered. While in some cases food provisioning leads to increase in breeding activity and survival, especially during periods of limited resource availability^11^, the majority of studies report that food-provisioning used by the tourism industry can have detrimental effects on wildlife^10,12–15^ and in particular when targeting top predators^16^. For example, food-provisioning can cause disruptions of natural behavioral patterns^10,14,17^, increased inter- and intra-specific aggression^18^ and changes in residency patterns and home range size^11,19^ that can lead to trophic cascade events and modified species assemblage^20,21^. Some studies also report that feeding wildlife associated with tourism frequently increases stress, injury, disease transmission and/or malnutrition of the animals^22,23^. In the case of cetaceans, provisioning has also been linked to unnatural behaviors that can be socially learned, such as patrolling, scavenging, depredation and begging^24,25^. In turn, these behaviors increase the exposure of individuals to vessels and fishing gear, leading to greater risk of collision and entanglements^13,24,26^. Repeated impact of anthropogenic disturbance on individual animals can lead to population level consequences due to cumulative exposure to disturbance or if a large enough proportion of the population is affected^27^.

Intentional (legal and illegal) food-provisioning of different dolphin species occurs in several locations worldwide, generally within coastal or easily accessible waters to humans. For example Amazon river dolphin (*Inia geoffrensis*) and tucuxi (*Sotalia fluviatilis*) in Brazil are fed from floatation platforms on the Amazon river that also allow tourists to swim with these wild dolphins^18^. Furthermore, common bottlenose dolphin (*Tursiops truncatus*) in Florida and Georgia, USA^13,24^, and indo-pacific bottlenose dolphin (*Tursiops aduncus*) and humpback dolphin (*Sousa sahulensis*) in Australia are fed by recreational boaters and fishermen^28^. In Australia, provisioning of free-ranging marine mammals is illegal under state and federal law (1998 Wildlife Conservation Notice), however it has been reported at several sites, including Cockburn Sound and Bunbury, Western Australia^28,29^. In Australia, dolphins are also food-provisioned as part of regulated, licensed provisioning programs. Four locations have been granted legal permission to conduct food-provisioning: Tangalooma and Tin Can Bay, Queensland, and Monkey Mia in Shark Bay and Bunbury, Western Australia (WA)^28,30^. Extensive research has been undertaken in Monkey Mia on the provisioned dolphin population, and this research has informed the WA state management authority to implement procedures that partially addressed the negative effects of food-provisioning^31^.

In Bunbury, 22 dolphins are currently, or have been, routinely fed as part of a state regulated provisioning program, conducted by the Dolphin Discovery Centre (DDC), a non-profit organization that also offers dolphin-watching and swim-with tours. Here, trained volunteers provision dolphins a small amount of fish (daily maximum of 500gr) which equates to approximately 4% of the estimated daily calorific requirement of an average non-lactating free-ranging dolphin^32,33^. The provisioning is conducted in a non-predictable manner, with the time of feeding dependent upon the dolphins, which is different to Monkey Mia where there are daily scheduled feeding sessions at specific time of day (for a detailed description of the dolphin food-provisioning procedure in Bunbury and Shark Bay see Mann *et al*.^34^). Moreover, differently from Monkey Mia, in Bunbury, the number and identity of provisioned dolphins is not restricted by law. In Bunbury, provisioned dolphins are fed at different rates depending on their visitation frequency, as such some dolphins are fed regularly because they regularly visit the interaction zone in front of the DDC while others only visit the interaction zone, and thus are fed, more sporadically.

The Bunbury dolphin population has been studied extensively and previous research has yielded valuable information on the population’s abundance^35,36^, social structure^37^, ranging patterns^38,39^, behavioral and foraging ecology^40,41^, population viability^42^ and genetic connectivity to other populations in south-western Australia^43^. In 2016, Manlik *et al*.^42^ predicted that the dolphin population was in decline, and would be reduced by 50% over the next twenty years. In light of this, it is important to determine any potential impacts of food-provisioning on the Bunbury dolphin population.

In this study, we capitalize on ten years of systematic data collection coupled with long-term historical data available from the DDC and explore (i) whether food-provisioning affects female reproductive success and; (ii) factors that may influence calf survival. We compared the mean reproductive success between provisioned and non-provisioned females, and further investigated whether food-provisioning, begging for food-handouts and location (sheltered vs open waters) influenced female reproductive success. We examined whether calf survival was influenced by maternal provisioning and begging status, birth order, preferred location and extreme climate fluctuations (El Niño Southern Oscillation (ENSO) phases). Concerns for the effect of food-provisioning on the reproductive success of female dolphins have previously been raised, based solely on anecdotal observations^34^. The present study provides the first empirical data regarding an actual negative impact of this practice on reproductive success and calf survivorship. Moreover, results from this study provide pertinent biological information to management agencies for informed decision making to ensure the long-term sustainability of the dolphin population and, in turn, the local tourism industry in a region of human population growth and infrastructure development.

## Methods

### Study site and data collection

**Figure 1:**
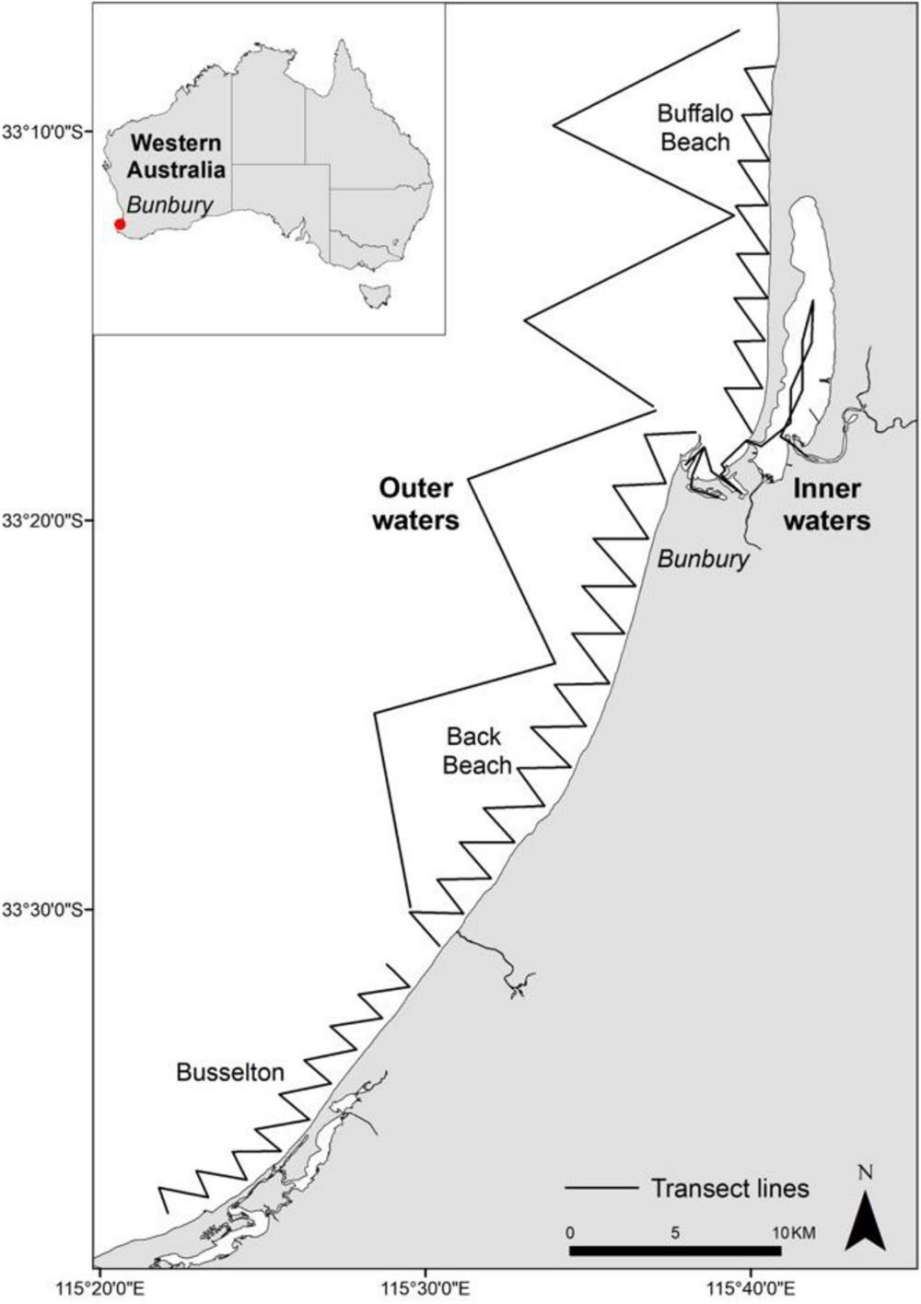
Study area off Bunbury, Western Australia. The insert map includes the transect routes followed during boat-based data collection. Zig-zag lines represent transects run along Busselton, Back Beach and Buffalo Beach (collectively representing “Outer waters”).

Data on dolphin groups were collected in the coastal waters around Bunbury, Western Australia (33°200S, 115°380E), from 2007 to 2016. Dedicated boat-based transects were conducted year-round following three routes: Buffalo Beach, Back Beach and Inner waters (combined referred to as the ‘Inshore transect’ which included the sheltered waters of Koombana Bay and the Leschenault Estuary) and encompassed an area of 120 km^2^ (Fig. 1). From 2012, three additional transects routes were added: Buffalo Beach open, Back Beach open and Busselton, increasing the survey area by 420 km^2^. Data on dolphins were recorded at group level and included Global Positioning System (GPS) position, time of dolphin encounter, water depth, predominant group behavior (during the first five minutes of the encounter), and group size and composition. Photo-identification (photo-ID) images of dorsal fins were collected to allow individual identification (for a detailed description of data collection see Sprogis *et al*.^35^). Data were collected in accordance with the relevant guidelines and regulations imposed by Murdoch University and the Western Australia Department of Biodiversity, Conservation and Attractions (www.murdoch.edu.au; www.legislation.wa.gov.au).

Data on occurrences of dolphin begging behavior were recorded opportunistically during transects. Begging is a type of human-dolphin interaction in which dolphins elicit food handouts from humans by approaching humans on boats or at the shore, away from the provisioning area^24,44^. Begging behavior indicates that an individual is conditioned to accept food-handouts from humans. Further observational records of females with dependent offspring were collected by volunteers of the DDC during dolphin-watching operations. Volunteers from the DDC have also kept systematic records of dolphins visiting the provisioning site since 2000. Data recorded by the DDC volunteers included the identity of provisioned dolphins, amount and type of fish provisioned and the duration of each visit.

### Female reproductive success and calf survival

Long-term photo-ID capture-recapture data from boat-based transects and DDC observational data were used to quantify female reproductive and calf survival histories. Reproductive success was calculated for females that had given birth to at least one calf and been sighted on >10 occasions between 2007 and 2016. Reproductive success of each female was calculated as a rate based on the number of calves that survived to 3 years of age in relation to the number of years in which a female was reproductively active^45^. Bottlenose dolphin calves are nutritionally dependent on their mother for a minimum of 1–2 years but weaning time varies between populations and individuals^12^ and can last for as long as eight years^12^. Date of weaning was estimated by taking the mid-point between the date of the last recorded infant position and the first sighting of the mother without the calf^46,47^. In Bunbury, the average weaning age is 2.93 years (calculated based on 54 calves from 46 mothers for which date of birth and date of weaning were known). Thus, for the purpose of this study, we considered three years of age as the minimum weaning age^48–50^. A calf was also considered weaned if it was replaced by another dependent calf^51^ and then sighted post-weaning. A calf was considered deceased if it was less than three years old at the time of its last sighting with its mother and the mother had been subsequently observed at least three times without the calf^51^. Calves whose weaning status was unknown were excluded from the analyses. Years of active reproduction were calculated for each female from the year of its first known birth to 2016 (included) or until the birth of her current dependent calf. If a female had not been sighted during three or more years, those years were excluded from the analyses as a calf could have been born and weaned during that time period without being recorded.

### Maternal characteristics

A female was considered conditioned to human food hand-outs and labelled as ‘provisioned’ if it had been fed by the DDC volunteers on at least five occasions between 2000 and 2016. To the extent of our knowledge, there are no references in the published literature regarding the length of time that it takes to condition a free-ranging dolphin to accept food rewards from humans. In the absence of references and to maximize the sample size, we selected five feeding events as a threshold to categorize a dolphin as being’provisioned’. To be more conservative with regards to the definition of a provisioned animal, the threshold could be raised. However, to reduce the current sample size by one dolphin would require the threshold to be set at 150 events and we can presume that consistent changes in the dolphins’ behavior will occur well before this point. Further, when this new threshold was tested, there was no change in the results of the analysis (see Supplemental material). Please note that in regards to provisioned females, we were able to estimate reproductive success for only 8 (out of 9) individuals and the minimum number of feeding events among those was 11 (Table 1). Additionally, we used the total number of provisioning events as an explanatory variable in our models to address the concern that females that were provisioned to a greater extent may be more adversely affected. No distinction was made between dolphins that were currently provisioned and dolphins that were provisioned in the past.

**Table 1:** Number of provisioning and begging events for reproductive females which begged and/or were provisioned at least once. Please note that not all provisioned females listed in the table have been used in our analyses.

Individual	Reproductive success	Provisioning events	Begging events
Bignick	0.14	394	2
Cracker	0.08	160	2
Key	NA*	15	2
Kwilina	0.12	1	0
Fence	0.11	11	0
Flattop	0.25	2	5
Leeuwin	0.25	0	1
Levy	0.12	2512	19
Lumpy	0.13	233	8
Mars	0.2	0	2
Mrs Iruka	0.25	0	26
Nicky	0.14	436	0
Shanty	0.10	1018	46
Tangles	0.25	466	0
Tipex	0.28	1	1
Wave	0.28	0	1

Reproductive success was calculated as a rate based on the number of weaned calves (calves that survived till three years of age) in relation to the number of years in which a female was reproductively active. *Reproductive success of Key was not calculated as her only calf was still dependent at the time of the study.

A female was defined as a begging dolphin if it had been observed begging at least once during the study period (from 2007 to 2016, Table 1). A similar definition was used by Finn *et al*.^29^ who classified a ‘conditioned’ dolphin (aka. a begging individual) from the first observation of the individual exhibiting begging behavior. Also, dedicated focal follow observations conducted in 2017–2018 suggest that individuals who exhibit begging are fairly consistent in this behavior (Senigaglia *et al*. unpublished data). Maternal age and experience can also influence calf survival. A recent study from Karniski *et al*. (2018) showed that calves of younger mothers have higher survival rates^46^. Reliable data on maternal age were insufficient to explore its effect on calf survival but birth order is related to maternal age and early calves have higher chances to survive to weaning age than following ones^46^. We categorized each calf as either the first recorded (aka earliest born according to our records) or not, based on combined data from ecological surveys and citizen science data collected by the volunteers of the DDC since 1990. We then used this binomial variable (here and after called “birth order”) as a predictor in our models of calf survival. While we cannot exclude that previous calves went undetected, our classification is a good approximation and allows us to partially explore the effect of maternal ageing on calf survival.

### External explanatory variables

We modelled the effect of climatic fluctuations on calf survival because reproduction in dolphins is particularly sensitive to environmental changes^52^. ENSO determines climate anomalies that affect rainfall and sea surface temperature (SST)^53^ that can affect neonate survival due to the limited thermal tolerance of calves^12,54,55^. ENSO cycles consist of three phases: La Niña, El Niño, and Neutral. We assigned ENSO phases based on the monthly Southern Oscillation Index (SOI) as in Sprogis *et al*.^39^. We obtained SOI monthly values from the Bureau of Meteorology and averaged the values across the year of birth for each calf, assigning an ENSO event to each year from 2007–2016 (events assigned as in Appendix 1 in Supplemental Material).

We also modelled the effect of location on calf survival. Bottlenose dolphins in Bunbury are exposed to various sources of human activities (other than provisioning), including recreational and commercial vessels^56^. Different locations within the study area have varying levels of anthropogenic disturbance (lower in open waters and higher in sheltered waters, Fig. 1) (Symons pers. comm.) and shark predation risk^57^. We assigned location based on where individual females were primarily sighted (>50% of sightings), considering the sheltered waters of Koombana Bay, the Leschenault Estuary and the Leschenault Inlet as “inner waters” and the coastal and open waters along Busselton, Buffalo beach and Back Beach as “outer waters” (Fig. 1).

## Analyses

We compared the mean reproductive success of provisioned and non-provisioned females (n = 55) using Bayes factor. We then used generalized linear models (GLMs) to determine the influence of food-provisioning, begging and location on female reproductive success. We further used GLMs to explore the influence of maternal characteristics (provisioned and begging status), birth order and environmental variables (habitat type and ENSO phase) on calf survival. Models were constructed by selecting explanatory variables based on biological meaning. Collinearity among explanatory variables was tested by calculating the variation inflation factor (VIF) using the package car in R v3.4.2 software^58^. We used Akaike’s Information Criterion (AIC) for model selection favoring the model with the lowest AIC^59–61^. We also provided BIC value^50^ and interpreted as further support to the best fitting model when AIC and BIC were in accordance. In the case when AIC and BIC were discordant in choosing the best fitting model, we interpreted as a sign of greater uncertainty. We also calculated Akaike weights to give a measure of strength of each model. Model residuals were visually examined to ensure lack of patterns due to unexplained variance or violation of independence^62^, whereas leverage and Cook’s distance were calculated to identify influential data points and outliers. All the analyses were run in R v3.4.2 software^58^.

### Female reproductive success

The Bayes factor computes the probability of a hypothesis being true conditionally on the empirical data and based on some prior distribution over the parameters, given the formula^63^.

$$\frac{\Pr \,(\mathrm{H1}|\mathrm{data})}{\,{\rm{\Pr }}\,(\mathrm{H0}|\mathrm{data})\,}=\frac{f({\rm{data}}|{\rm{H}}1|)}{f({\rm{data}}|{\rm{H}}0)}\times \frac{\Pr ({\rm{H}}1)}{\Pr ({\rm{H}}0)}$$

where Pr(H_1_)/Pr(H_0_) are the prior distribution while the ratio of probability of the two hypotheses *f* (data|H_1_)/*f* (data|H_0_) is the Bayes factor^64^, that could be directly interpreted as evidence in support of a hypothesis given the data^63^. In our case H_1_ corresponded to the hypothesis of a true difference in the mean reproductive success of provisioned and non-provisioned females, while H_0_ indicated the null hypothesis of no difference between the two means, given the data. The analyses were run using the package Bayes factor^65^ in R. This package assumes normal distribution (Gaussian) of the response variable (aka reproductive success) and uses non-informative Jeffreys priors on the variance (mean = 0, scale = sqrt(2)/2) and a Cauchy prior (mean = 0, scale = 2.5) on the standardized effect size, as suggested for routine use in Rouder *et al*.^63^. For Bayes factor interpretation, we referred to the scale provided in Rouder *et al*.^63^ and based on Jeffreys H.^64^: odds > 3 are considered “some evidence,” odds > 10 are considered “strong evidence,” and odds > 30 are considered “very strong evidence” for one of the hypothesis^63^. To ensure robustness of our results, we randomly selected eight non-provisioned females and used Bayes factor to compare their reproductive success with the rest of the dataset (including provisioned females, n_total_ = 63). We then bootstrapped our results 1,000 times and compared the mean Bayes factor, obtained from the bootstrap, with the observed (all non-provisioned females vs. provisioned females) Bayes factor. We expected the randomly selected non-provisioned females to not differ significantly (odds~0) from the rest of the data set, whereas we expected the provisioned females to differ significantly from the non-provisioned females (odds > 3).

To explore other factors that could influence female reproductive success, we developed GLMs using the number of weaned calves as the response variable and a Poisson distribution and a log link function (for count data). The number of years of reproduction was included as an offset of the model. Using the offset to control for the number of years of reproduction gives an estimated reproductive success comparable to our calculated ones, thus we used the same terminology. Explanatory variables included: provisioning and begging status (coded as binomial variables), the number of provisioning events, the number of begging events and the location (sheltered versus open waters). There was no collinearity among explanatory variables (VIF values < 3). A backward stepwise procedure was used to sequentially remove variables and interactions between variables from the full model based on their biological significance. Overdispersion was tested by dividing the squared value of the Pearson’s residuals by the total number of observation minus the number of coefficients estimated by the model. Correction for overdispersion was not necessary.

### Calf survival

Two sets of GLMs were developed to explore the effect of external and maternal variables on calf survival to years one and three (weaning age) respectively. Calf survival was modelled as a Bernoulli response variable (yes or no). Explanatory variables included: mother provisioning status (binary) and begging status (binary), preferred location (inner versus outer waters), ENSO phases in the year of birth of the calf (assigned as La Niña, El Niño and Neutral based on averaged SOI value for the year) and whether the calf was the earliest recorded born (binary). In regards to begging status, this was assigned regardless of the time of the first recorded begging event, assuming that a female who displayed begging behavior in the data collection period (2007–2016) was already familiar with it. We were not able to include maternal age as a predictor of calf survival due to lack of data. Instead, we used two different approaches to try to account for maternal age. First, we included birth order as a predictor assuming that earlier calves would have younger mothers. Second, we modelled the very same response and explanatory variables in generalized mixed effect model (GLMM) using the years of reproduction available per female as a random effect controlling for the fact that older females would have more years of reproduction. Please note that we have no information on the birth or fate of calves born to provisioned females prior to them being provisioned. Explanatory variables did not show collinearity (VIF values < 3).

## Results

### Effect of food-provisioning on female reproductive success

**Figure 2:**
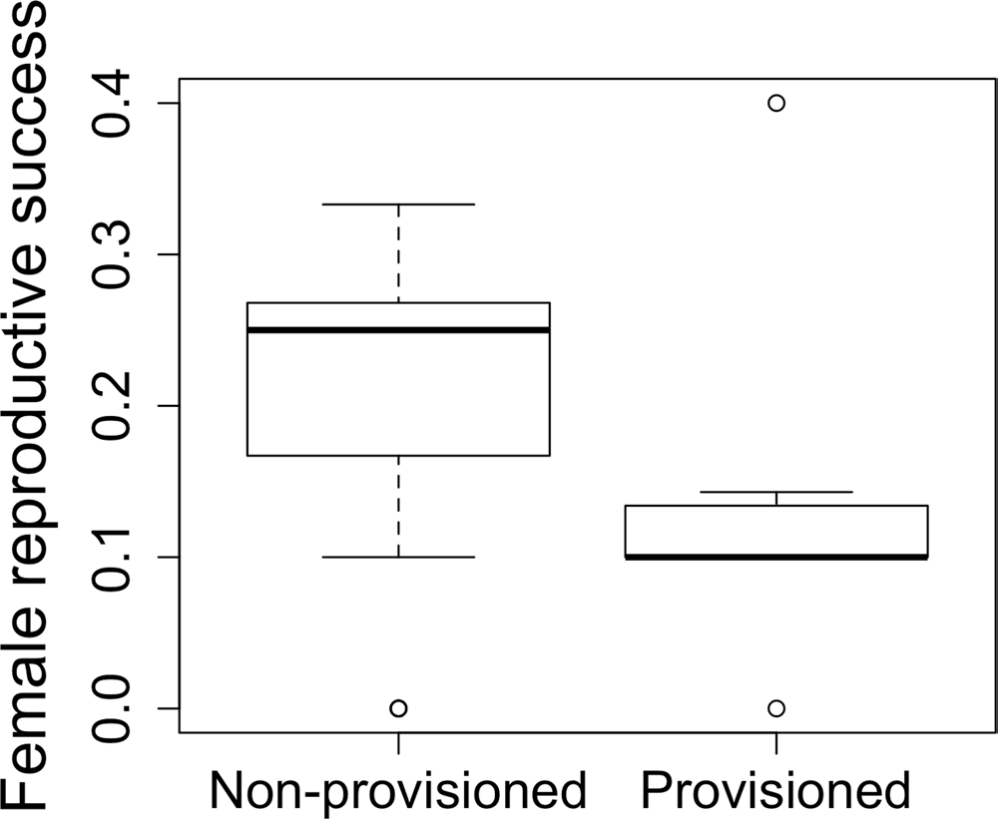
Boxplot of the reproductive success of non-provisioned (*n* = 55) and provisioned (*n* = 8) females. Female reproductive success was calculated as a rate based on the number of weaned calves in relation to the number of years in which a female was reproductively active. The solid black lines represent the median values while the lower and upper end of each box represent the lower and upper 75% quantiles, respectively. The whiskers (dotted lines) represent the range of the data.

We calculated the reproductive success of 63 females, eight of which had been or were currently being provisioned by the DDC. The maximum number of weaned calves per female was four (median = 1; SD = 0.89) and the median number of weaned calves was two (SD = 0.47). The Bayes factor analyses provided some evidence in favor of the hypothesis of a true difference in reproductive success between provisioned and non-provisioned females with the former ones having, on average, a lower reproductive success (Bayes factor = 6.2; Fig. 2). The bootstrapped mean Bayes factor, which compared eight randomly selected non-provisioned females with the rest of the females, was considerably lower (Bayes factor = 0.6) than the one obtained from the comparison of “true” provisioned vs non-provisioned females. This gives further support to a true difference between provisioned and non-provisioned females, as opposed to by chance, despite the low threshold used for defining provisioned females and the low sample size. In our analyses of reproductive success, AIC and BIC values both indicated the model with only provisioning as explanatory variable as the best fitting (model 1 in Table 2), with food-provisioned females having lower reproductive success than their non-provisioned counterparts (Wald = −1.703; P value = 0.0886). Non-provisioned females had double the reproductive success (0.20, CI: 0.18–0.23, weaned calves per year of reproduction) of provisioned females (0.11, CI: 0.06–0.17, weaned calves per year of reproduction). The model including provisioning events (number of times that a female was provisioned by the DDC) scored an AIC value of <2 from the most parsimonious model (model 2 in Table 2). A difference in AIC and/or BIC values of <2 is generally interpreted as equal support for the two models^59^, thus the frequency of provisioning might also have an effect on females’ reproductive success (Wald = −1.069; P value = 0.285). However, Akaike’s weight for model 1 is considerably higher (w_i_ = 0.44, Table 2) than for model 2 (w_i_ = 0.17, Table 2), suggesting that provisioning frequency is less influential on reproductive success than provisioning status. Our data also show a slight correlation between provisioning events and the number of begging events recorded (F_1,29_ = 3.64, P value = 0.06), however begging *per se* did not significantly affect reproductive success (Wald = −0.32, P value* = *0.749) and the model containing begging as sole explanatory variable explained minimal deviance within our data (model 6 in Table 2).

**Table 2:** Model selection results of GLMs of female reproductive success (RS) as a function of preferred location (inner vs outer waters), provisioning status, begging status and the number of times a female was provisioned (provision events) and observed begging (begging events).

Model	Variables	AIC	∆AIC	*w*_*i*_	BIC	∆BIC	d.f.
1	**RS ~ provisioning + offset**	**157**.**61**	**0**.**00**	**0**.**44**	**161**.**90**	**0**.**00**	**61**
2	RS ~ provision events + offset	159.48	1.87	0.17	163.77	1.87	61
3	RS ~provisioning + provision events + offset	159.61	1.99	0.05	166.08	4.14	60
4	RS ~ begging events + offset	160.36	2.74	0.11	164.64	2.74	61
5	RS ~location + offset	160.49	2.88	0.10	164.78	2.88	61
6	RS ~ begging + offset	160.83	3.21	0.08	165.11	3.21	61
7	RS ~ begging + begging events + offset	162.34	4.72	0.01	168.77	6.87	60
8	RS ~ provisioning + provision events + begging + begging events + offset	163.19	5.58	0.00	173.91	12.01	58
9	RS ~ begging * begging events + offset	164.01	6.39	0.00	172.58	10.68	59
10	RS ~ provisioning + provision events + begging + begging events + location + offset	165.13	7.52	0.00	177.99	16.09	57
11	RS ~provisioning*provision events + offset	167.03	9.41	0.00	182.03	20.13	56
12	RS ~ 1	168.98	11.36	0.00	171.12	9.22	62

The offset represents the number of years for which reproduction data were available, calculated from the birth year of the first known calf of each female. Models are listed in ascending order of AIC value. AIC and BIC values are provided with ∆AIC and ∆BIC (difference in AIC and BIC values compared to the most parsimonious model) (number 1, highlighted in bold). w_i_ = Akaike weight values are provided.

### Effect of food-provisioning on calf survival

Our dataset comprised of a total of 152 calves born between 1996 and 2015 to 68 mothers, however sufficient data on calf survival histories were available for only 145 calves born to 63 mothers. Of these, 85% (N = 123) survived their first year and 77% (85 out of 129 of know-fate calves) were successfully weaned. However, only 38% of the calves born to provisioned mothers (N = 11 of 29 of known fate) survived to three years of age. Generalized mixed effect models revealed low variance (σ^2^ = 0.05) associated with the random effect, years of reproduction, meaning that maternal age might not be a source of variance in calf survival. According to the best fitting model, containing both provisioning and birth order as a predictor, calves of provisioned females had a lower survival rate to weaning age and earlier born calves had a slightly higher survival rate than subsequent offspring (model 1 in Table 3). However, the model containing only provisioning status as predictor, had equal support of the most parsimonious one (difference in AIC values < 2). The best fitting model according to BIC included only provisioning as explanatory variable (model 2 in Table 3) confirming the influence of provisioning on calf survival. None of the external variables considered (ENSO phases and location) were included in the most parsimonious models (Table 3). The results from analyses of first year survival mirrored the ones of survival to weaning age in highlighting the influence of provisioning on calf survival but the signal was not as strong with 7 models, including the full and the null model, within 2 points of AIC (models 1 to 7 in Table 4). The absolute best fitting model according to AIC included both provisioning and year of birth (model 1 in Table 4) however the small difference in AIC values across models suggests that other predictors, including environmental variables, might be influential in determining first year survival of the calves.

**Table 3:** Model selection results of GLMs where calf survival to year three (weaning age) is modelled in relation to their preferred location (inner vs outer waters), the provisioning and begging status of the mother, the birth order (whether the calf was the first recorded or not) and ENSO events during the year of birth (La Niña, El Niño, Neutral).

Model	Variables	AIC	∆AIC	w_*i*_	BIC	∆BIC	d.f.
**1**	**weaned ~ birth order + provisioning**	**137**.**24**	**0**.**00**	**0**.**46**	**145**.**37**	**2**.**03**	**108**
2	weaned ~ provisioning	137.91	0.67	0.33	143.33	0.00	109
3	weaned ~ ENSO + provisioning	140.78	3.54	0.07	151.62	8.28	107
4	weaned ~ birth order	141.10	3.86	0.06	146.52	3.18	109
5	weaned ~ birth order + provisioning + location + ENSO + begging	143.59	6.35	0.01	162.56	19.22	104
6	weaned ~ 1	144.65	7.41	0.01	147.36	4.02	110
7	weaned ~location	145.03	7.79	0.00	150.45	7.12	109
8	weaned ~ begging	145.44	8.19	0.00	150.86	7.52	109
9	weaned ~ ENSO	146.08	8.84	0.00	154.21	10.87	108
10	weaned ~ ENSO + location	147.55	10.31	0.00	158.39	15.05	107
11	weaned ~ ENSO + begging	147.48	10.24	0.00	158.32	14.98	107

Models are listed in ascending order of AIC value. AIC and BIC values are provided alongside with ∆AIC and ∆BIC (difference in AIC and BIC values compared to the most parsimonious model) (number 1, highlighted in bold). w_i_ = Akaike weight values are provided.

**Table 4:** Model selection results of GLMs where newborn survival in the first year of life (S) is modelled in relation to their preferred location (inner vs outer waters), the provisioning and begging status of the mother, the birth order (whether the calf was the first recorded or not) and ENSO events during the year of birth (La Niña, El Niño, Neutral).

Model	Variables	AIC	∆AIC	w_i_	BIC	∆BIC	d.f.
**1**	**S~ birth order + provisioning**	**113**.**79**	**0**.**00**	**0**.**17**	**122**.**32**	**3**.**57**	**124**
2	S ~ provisioning	114.09	0.30	0.14	119.78	1.03	125
3	S ~ birth order	114.26	0.47	0.13	119.95	1.20	125
4	S ~ ENSO + provisioning	114.40	0.61	0.12	125.78	7.03	123
5	S ~ ENSO	115.01	1.22	0.09	123.55	4.80	124
6	S ~location	115.44	1.65	0.07	121.13	2.38	125
7	S ~ birth order + provisioning + location + ENSO + begging	115.50	1.71	0.07	135.41	16.66	120
8	S ~ 1	115.90	2.11	0.06	118.74	0.00	126
9	S ~ ENSO + location	116.28	2.49	0.04	127.66	8.91	123
10	S ~ ENSO + begging	116.81	3.02	0.03	128.19	9.44	123
11	S ~ begging	117.89	4.10	0.02	123.58	4.83	125

Models are listed in ascending order of AIC value. AIC and BIC values are provided alongside with ∆AIC and ∆BIC (difference in AIC and BIC values compared to the most parsimonious model) (number 1, highlighted in bold). w_i_ = Akaike weight values are provided.

## Discussion

Population viability in long-lived species is determined by survival and reproductive rate^42^. In Bunbury, a previous study  suggests that the local dolphin population is particularly sensitive to disruption of female reproductive output and the population is forecasted to decline in abundance at the current level of reproductive output^42^. The study conducted by Manlik *et al*.^42^ defined reproductive rate as the ratio between the number of reproductive females and the total number of adult females over a three-year period and thus assumed equal female reproductive success within the population. However, our data showed that provisioned females have a lower reproductive success compared to non-provisioned females. After accounting for the number of years of reproduction in the two groups, provisioned and non-provisioned females produced similar number of calves (mean = 3.1 and 2.9 respectively) but, on average, a provisioned female weaned half as many calves as a non-provisioned female. Other factors that could influence individual reproductive success, such as the location (inner vs outer waters) and begging behavior (reflecting a limited wariness towards vessels), were not found to be significant in our analyses.

We found that the first-year calf survival rate for the entire population, provisioned and non-provisioned included, (85%) was consistent with those calculated for other populations (76% in Monkey Mia, WA^12^, 86% in Mikura Island, Japan^66^, 81% in Sarasota, FL^67^ and 86% in Doubtful Sound, NZ, from ‘94 to ’99^55^). Pre-weaning (age <3 years) mortality rate (n_total_-n_deceased_) of calves of non-provisioned females (26%) was also similar to other populations (17% in the North Sea^68^; 20% in Fjordland, NZ^69^) but much lower than that reported for dolphins in Shark Bay, WA (40–46%^12^). However, when considering only calves of provisioned females, pre-weaning mortality rate increased to 61%, similar to Monkey Mia in 1994 (62%^12^) pre-provisioning protocol. In Monkey Mia, the dolphin feeding program has been monitored by researchers since 1984 and a review of the practice in 1994 revealed the considerably lower reproductive success of provisioned females compared to their non-provisioned counterparts^12^. Following these results, the governmental agency responsible for the provisioning program (currently the Department of Biodiversity, Conservation and Attractions) took decisive management actions restricting the daily amount of fish fed to the dolphins, limiting the time spent at the feeding area and the number of individuals fed^34^. The provisioning protocol in Bunbury is based on the new management strategy adopted in Monkey Mia and has further restrictive guidelines in terms of the maximum daily amount of fish that can be provisioned to individual dolphins^34^. Yet, our results show a lower reproductive success and calf survival of provisioned females comparable to the calf mortality recorded in Monkey Mia before the government intervention.

Maternal characteristics other than provisioning status, such as age, experience and behavior are known to influence calf survival. A recent article by Karniski *et al*.^46^ highlighted a linear correlation between calf survival and maternal age, with younger mothers producing more successful calves. Our data did not allow for a comprehensive analysis of reproductive senescence since estimates of females’ age were not always known. However, we explored whether the first recorded calves had a different survival probability than subsequent offspring assuming that birth order reflect maternal age^46^ (aka the earlier the calf, the younger the mother). Our results show that first recorded calves had a higher survival probability than subsequent offspring, however the support for this predictor was weak. Similar results have been found in Shark Bay^46^ while, research on other species, such as killer whales (*Orcinus orca*), found that calves from oldest mothers have higher probabilities of survival but birth order was not influential^70^. In our study, data on birth order relied also on citizen science and we cannot exclude that calf births might have been misreported by the DDC and that individuals that reside within the inner waters of Koombana Bay and the Leischenault Estuary might have been more frequently sighted by the DDC compared to others individuals. Thus, the influence of birth order on calf survival should be interpreted cautiously in our study.

The strong bond between bottlenose dolphin mothers and calves implies that maternal behavior is likely to influence calf survival. In Sarasota, Florida, common bottlenose dolphins that engage in begging behavior are at higher risk of boat strikes, propeller cut injuries and fishing gear entanglements^13^. Based on this, a begging mother in Bunbury might put her calf at greater risk of injuries, therefore, we also explored whether a mother’s behavior towards vessels might influence her calf survival. We found a correlation between provisioning and begging events suggesting that females that were frequently provisioned by the DDC were also observed to beg around boats more often. Calves from provisioned animals are particularly vulnerable to adopt such a behavior due to strong vertical transmission of foraging techniques^31,71^. While a direct causal link between begging and provisioning cannot be made, our data suggests that calves of provisioned animals are more likely to approach boats and perform begging behavior (Senigaglia, unpublished data), that in turn may affect injury risk and survival^13^. Good maternal skills could also be acquired, either transmitted from mother to daughter or learnt socially from more experienced female companions^72^. However, social network and social behavior comparisons of provisioned vs non-provisioned animals were outside of the scope of this study but should be addressed in future research.

Calf survival can also be influenced by the surrounding environment^12,68^. Neonates have limited thermal tolerance and considerable reductions in SST can decrease the ability of a calf to survive to weaning age^54,55^. Extreme climate fluctuations such as ENSO events can cause variations in SST such that can affect calf body condition^55^. Moreover, environmental fluctuations can affect prey availability which, in turn, might affect female’s energetic budget during lactation and hinder her ability to raise a healthy calf^32,73^. Our results do not support the hypothesis that extreme climatic fluctuations significantly affect calf survival to weaning age. However, results from the analyses of survival to 1 year of age revealed greater uncertainty and environmental characteristics and climatic fluctuations might have an influence on newborn survival (survival to 1 year of age).

Calf survival and female reproductive success can be affected by predation risk that could vary among locations^74^. The main predators of bottlenose dolphins in Bunbury are sharks of various species. Overall, non-lethal predation of sharks affects 18.6% of non-calves in Bunbury (which is lower than in Shark Bay where 74.2% of non-calves present evidence of non-lethal shark attack^57^) but predation probability varies among areas with a higher proportion of non-lethal attacks detected in inner waters^57^. However, dolphins that share the same home range and habitat use are subjected to the same level of predation risk. Our results do not show differences in reproductive success or calf survival to weaning age between locations (inner vs outer waters). It is likely that predation would greater affect calf survival during the first year of age (for which our results show a greater degree of uncertainty).

Anthropogenic disturbance (e.g. boating, shipping, noise, pollution) could also affect calf survival. In Bunbury, the highest level of recreational boating activities occurs during the austral summer and autumn, due to a substantial increase in the number of recreational boats (Symons pers. comm.). This time period coincides with the dolphins’ main calving season^37^. Anthropogenic effects could vary across different areas but can be considered homogeneous among dolphins living in the same area. We attempted to account for differences in human pressures by incorporating the effects of two type of locations, inner and outer waters. We assumed a greater impact of anthropogenic disturbance closer to the harbor and coastal developments located in the inner waters^75–77^ (Symons pers. obs). Our results showed no effects of location on reproductive success or calf survival, however, the ongoing expansion of Bunbury waterfront and harbor is a reason for concern. The plan for the harbor expansion includes the construction of 450 additional boat pens and the redevelopment of the DDC that will increase tourism activities along with the anthropogenic pressure on this coastal dolphin population during and after construction works^78–80^. In summary, while the mechanism that links provisioning to reduced reproductive success and calf survival is still unknown, our data shows a difference in reproductive success between provisioned and non-provisioned females. We do not exclude that other factors might affect reproductive success and calf survival, but our data indicates provisioning as the most significant among the variables explored.

## Conclusion

Long-term biological effects of nature-based tourism on cetaceans have been suggested in several studies^81–83^, however empirical evidence has been limited due to the lack of available long-term datasets^74,84^. By capitalizing on a decade of systematic data collection, we documented the difference in female dolphin reproductive success and calf survival between females targeted by a nature-based tourism activity (food-provisioning) and non-provisioned females. The provisioning program at the DDC is state licensed under conditions that aim to minimize long-term impacts. Additionally, the DDC has developed self-regulatory measures to ensure sustainability of the provisioning practice, based on the revised feeding protocols adopted in Monkey Mia. Yet, despite the self-regulations in place, our results show that calves of provisioned females have an unusually high mortality rate, similarly to the calf survival of provisioned females in Monkey Mia before the management interventions. Since calves that die before weaning are a missed recruit for the population, our results show that dolphin-focused tourism, in this case limited to food-provisioning, has the potential to worsen the forecast made by Manlik *et al*.^42^ and exacerbate the risk for this population to decline. Future studies should focus on understanding the mechanism that ultimately led to a decreased reproductive success of provisioned females (e.g. reduced maternal care or differences in behavioural budgets between provisioned and non-provisioned dolphins). A good understanding of the mechanistic process will aid in determining the most suitable management actions to reduce the impact and minimize the effect on the socio-economic side of eco-tourism. In turn, this will ensure a sustainable nature-based-tourism industry that will benefit both the dolphin population and the local economy that relies on dolphins as the main tourist attraction.

### Ethical approval

This research has been conducted with the approval from the Murdoch University Animal Ethics Committee and under the permit (SF005811, SF007986, SF008624, SF009734, SF010223 and SF10738) issued by the Western Australia Department of Biodiversity, Conservation and Attractions (Previously Department of Environment and Conservation and Department of Parks and Wildlife). This paper represents HIMB and SOEST contribution numbers 1760  and 10715, respectively.

## Supplementary information

Supplementary information for: Food-provisioning negatively affects the reproductive success of female bottlenose dolphins and calf survival
