# Author Correction: Food-provisioning negatively affects calf survival and female reproductive success in bottlenose dolphins

**DOI:** 10.1038/s41598-020-75955-0

**Published:** 2020-10-28

**Authors:** V. Senigaglia, F. Christiansen, K. R. Sprogis, J. Symons, L. Bejder

**Affiliations:** 1 Aquatic Megafauna Research Unit, Centre for Sustainable Aquatic Ecosystems, Harry Butler Institute, Murdoch University, Murdoch, 6150 Australia; 2 Environmental and Conservation Sciences, Murdoch University, Murdoch, 6150 Australia; 3 Aarhus Institute of Advanced Studies, Høegh-Guldbergs Gade 6B, 8000 Aarhus, Denmark; 4 Zoophysiology, Department of Bioscience, Aarhus University, 8000 Aarhus, Denmark; 5 Hawaii Institute of Marine Biology, University of Hawaii at Manoa, Kaneohe, HI 96744 USA

Correction to: *Scientific Reports* 10.1038/s41598-019-45395-6, published online 20 June 2019

This Article contains errors.

In the Introduction,

“For example Amazon river dolphin (*Inia geoffrensis*) and tucuxi (*Sotalia fluviatilis*) in Brazil are fed from floatation platforms on the Amazon river that also allow tourists to swim with these wild dolphins^18^”.

should read:

“For example Amazon river dolphin (*Inia geoffrensis*) in Brazil are fed from floatation platforms on the Amazon river that also allow tourists to swim with these wild dolphins^18^”.

Additionally, in the Analyses section under subheading ‘Female reproductive success’,

“where Pr(H_1_)/Pr(H_0_) are the prior distribution while the ratio of probability of the two hypotheses *f* (data|H_1_)/*f* (data|H_0_) is the Bayes factor^64^, that could be directly interpreted as evidence in support of a hypothesis given the data^63^.”

should read:

“where Pr(H_1_)/Pr(H_0_) are the prior distribution while the ratio of probability of the two hypotheses *f* (data|H_1_)/*f* (data|H_0_) is the Bayes factor^64^, that could be directly interpreted as a measure of the evidence in support of H_1_ relative to H_0_ given the data and priors (aka the odds of H_1_ relative to H_0_; ^63^).”

Finally, in the Legend of Figure 2,

“The whiskers (dotted lines) represent the range of the data.”

should read:

“The whiskers (dotted lines) indicates data variability outside the upper and lower quartile.”

