# Supplementary material for: Food-provisioning negatively affects calf survival and female reproductive success in bottlenose dolphins
Source: Sci Rep. 2019 Jun 20;9:8981. doi: 10.1038/s41598-019-45395-6 (PMC6586622; doi:10.1038/s41598-019-45395-6)
Supplement: Supplementary file 1 — Supplementary information for: Food-provisioning negatively affects the reproductive success of female bottlenose dolphins and calf survival [file 41598_2019_45395_MOESM1_ESM.docx]

**Supplementary material for: Food-provisioning negatively affects calf survival and female reproductive success in bottlenose dolphins**

Senigaglia V., Christiansen F., Sprogis K.R., Symons J., Bejder L.

**Testing the threshold for minimum number of provisioning events:**

To the best of our knowledge, there are no references in the literature regarding how long it takes to condition a wild dolphin to accept food rewards from human. In many instances the behavioral conditioning process appears to be fast but there is no specific threshold for provisioning. In absence of further references and to maximize the sample size, we used 5 as a threshold to categorized a dolphin as “provisioned. To weight the sensitivity of our chosen threshold, we ran the same analyses on a modified dataset in which we defined as “provisioned” only females who were fed >150 times and we obtained identical results. This analysis lead to no significant difference (Table 2, Figure 2; Table 1 and Figure 1 refer to the original analyses and are provided for comparison). Thus, to be more conservative with regards to the definition of a provisioned animal, the threshold could be raised. However, to reduce the current sample size by one would require the threshold to be set at 150 events and we can presume consistent changes in the dolphins’ behavior will occur well before this point. Further, even when this new threshold was tested, there was no change in the results of the analysis. Therefore, increasing the threshold doesn’t affect our results, but instead would mask what happens at the lower range on the dolphins’ sensitivity to provisioning. In light of this, we feel that maintaining 5 feeding events as a threshold to categorized an animal as provisioned is a valid approach that allows to maximize sample size while obtaining unbiased results.

Figures

Figure 1. Boxplot of the reproductive success of non-provisioned (n=55) and provisioned (n=8) females. **A** **threshold of > 5 feeding events was used to define provisioned females.** Female reproductive success was calculated as a rate based on the number of weaned calves in relation to the number of years in which a female was reproductively active. The solid black lines represent the median values while the lower and upper end of each box represent the lower and upper 75% quantiles, respectively. The whiskers (dotted lines) represent the 95% and the 5% quantiles.

Figure 2. Boxplot of the reproductive success of non-provisioned (n=56) and provisioned (n=7) females. **A threshold of > 150 feeding events was used to define provisioned females.** Female reproductive success was calculated as a rate based on the number of weaned calves in relation to the number of years in which a female was reproductively active. The solid black lines represent the median values while the lower and upper end of each box represent the lower and upper 75% quantiles, respectively. The whiskers (dotted lines) represent the 95% and the 5% quantiles.

Tables

Table 1. Model selection results of GLMs of female reproductive success (RS) as a function of preferred location (sheltered vs open waters), provisioning status, begging status and the number of times a female was provisioned (provision events) and observed begging (begging events). The offset represents the number of years for which reproduction data were available, calculated from the birth year of the first known calf of each female. AIC and BIC values are provided with ∆AIC and ∆BIC (difference in AIC and BIC values compared to the most parsimonious model) (number 7, highlighted in bold). w_i_ = Akaike weight values are provided. **A threshold of > 5 feeding events was used to define provisioned females.**

| Model | Variables | AIC | ∆AIC | *w_i_* | BIC | ∆BIC | d.f. |
| --- | --- | --- | --- | --- | --- | --- | --- |
| 1 | RS ~ 1 | 168.98 | 11.36 | 0.004 | 171.12 | 9.22 | 62 |
| 2 | RS ~ provisioning + provision events + begging + begging events +location + offset | 165.13 | 7.52 | 0.000 | 177.99 | 16.09 | 57 |
| 3 | RS ~ provisioning + provision events + begging + begging events + offset | 163.19 | 5.58 | 0.001 | 173.91 | 12.01 | 58 |
| 4 | RS ~provisioning + provision events + offset | 159.61 | 1.99 | 0.055 | 166.08 | 4.14 | 60 |
| 5 | RS ~provisioning*provision events + offset | 167.03 | 9.41 | 0.000 | 182.03 | 20.13 | 56 |
|  | RS ~ begging + begging events + offset | 162.34 | 4.72 | 0.014 | 168.77 | 6.87 | 60 |
| 6 | RS ~ begging * begging events + offset | 164.01 | 6.39 | 0.002 | 172.58 | 10.68 | 59 |
| **7** | **RS ~ provisioning + offset** | **157.61** | **0.00** | **0.442** | **161.90** | **0.00** | **61** |
| 8 | RS ~ begging + offset | 160.83 | 3.21 | 0.088 | 165.11 | 3.21 | 61 |
| 9 | RS ~location + offset | 160.49 | 2.88 | 0.104 | 164.78 | 2.88 | 61 |
| 10 | RS ~ provision events + offset | 159.48 | 1.87 | 0.173 | 163.77 | 1.87 | 61 |
| 11 | RS ~ begging events + offset | 160.36 | 2.74 | 0.112 | 164.64 | 2.74 | 61 |

Table 2. Model selection results of GLMs of female reproductive success (RS) as a function of preferred location (sheltered vs open waters), provisioning status, begging status and the number of times a female was provisioned (provision events) and observed begging (begging events). The offset represents the number of years for which reproduction data were available, calculated from the birth year of the first known calf of each female. AIC and BIC values are provided with ∆AIC and ∆BIC (difference in AIC and BIC values compared to the most parsimonious model) (number 7, highlighted in bold). wi = Akaike weight values are provided. **A threshold of > 150 feeding events was used to define provisioned females.**

| Model | Variables | AIC | ∆AIC | *w_i_* | BIC | ∆BIC | d.f. |
| --- | --- | --- | --- | --- | --- | --- | --- |
| 1 | RS ~ 1 | 157.28 | 4.0 | 0.04 | 159.4 | 1.9 | 62 |
| 2 | RS ~ provisioning + provision events + begging + begging events +location + offset | 159.62 | 6.3 | 0.01 | 172.4 | 14.9 | 57 |
| 3 | RS ~ provisioning + provision events + begging + begging events + offset | 157.62 | 4.3 | 0.03 | 168.3 | 10.8 | 58 |
| 4 | RS ~provisioning + provision events + offset | 155.23 | 1.9 | 0.11 | 161.6 | 4.1 | 60 |
| 5 | RS ~provisioning*provision events + offset | 161.60 | 8.3 | 0.00 | 176.6 | 19.0 | 56 |
|  | RS ~ begging + begging events + offset | 157.33 | 4.0 | 0.03 | 163.7 | 6.2 | 60 |
| 6 | RS ~ begging * begging events + offset | 157.33 | 4.0 | 0.03 | 163.7 | 6.2 | 60 |
| **7** | **RS ~ provisioning + offset** | **153.24** | **0.0** | **0.30** | **157.5** | **0.0** | **61** |
| 8 | RS ~ begging + offset | 155.74 | 2.5 | 0.08 | 160.0 | 2.5 | 61 |
| 9 | RS ~location + offset | 155.75 | 2.5 | 0.08 | 160.0 | 2.5 | 61 |
| 10 | RS ~ provision events + offset | 154.59 | 1.3 | 0.15 | 158.8 | 1.3 | 61 |
| 11 | RS ~ begging events + offset | 155.51 | 2.2 | 0.09 | 159.8 | 2.3 | 61 |

**Appendix 1**

**SOI monthly values from the Australian Government Bureau of Metereology**
